# Problem Solved: An Interview with Sir Edwin Southern

**DOI:** 10.1371/journal.pgen.1003344

**Published:** 2013-03-14

**Authors:** Jane Gitschier

**Affiliations:** Departments of Medicine and Pediatrics and Institute for Human Genetics, University of California San Francisco, San Francisco, California, United States of America

Yes. In answer to your question, yes, this is an interview with *the* Southern, as in the eponymous blot. Devised in the mid-1970s, Southern's technique for transferring DNA from gels onto nitrocellulose paper allowed single-copy, eukaryotic genes to be discerned for the first time. It quickly became a mainstay for a generation of molecular biologists and spawned a template for mapping the human genome.

But the blot was no one-off for Southern, who is now entering his sixth decade at the bench. Working in Edinburgh in the Medical Research Council (MRC) Mammalian Genome unit, he was one of the first to sequence eukaryotic DNA and to appreciate the genetic architecture of satellite DNAs. He was also a strong and productive proponent of physical mapping of the human genome as a complement to the genetic map. In the late 1980s, while Professor of Biochemistry at Oxford, Southern conceived of oligonucleotide microarrays for DNA sequencing and was issued a patent for the invention. This award led to his founding of a small company, Oxford Gene Technology (OGT), and through successful lawsuits to defend his patent from infringement, to licensing the patent as a source of funding for two highly successful philanthropic trusts: the Edina Trust, which supports science education in the United Kingdom, and the Kirkhouse Trust, which develops disease- and pest-resistant legume crops in Africa and India.

I visited Southern in November during one of the wettest years in Oxford history, as this grey day sprinkled its contribution into the record books. We met at the Trinity Gate, where I was given shelter by the porter as Southern arrived under a large umbrella (Image 1). We headed for the tower, then up a flight of stairs to a cozy room for coffee and discussion. It was just as the reader might imagine: oak-paneled walls chock-a-block with portraits of former fellows, dons, and benefactors, upholstered chairs, rugs, and an attractive grandfather clock that allegedly chimes twice at 10 and, as we witnessed, 10 times at 11. Southern is soft-spoken, his voice low and gravelly, and I hung on every word.

**Figure:**
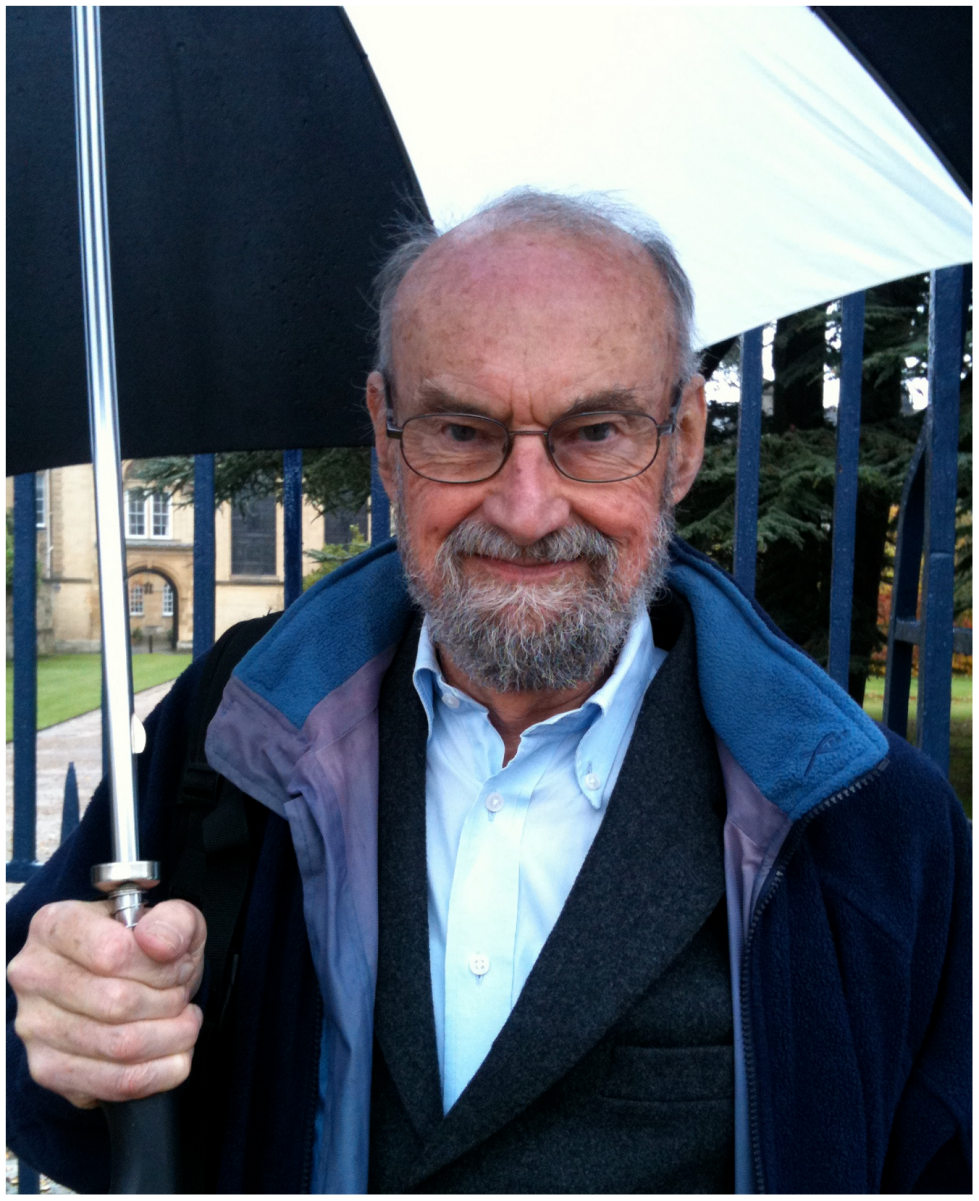
Sir Edwin Southern. Photograph by Jane Gitschier.

**Gitschier:** When you look back over the course of your research career, what would be—for *you*—the highlights?

**Southern:** I think that for me the thrills are really what happens in the lab. It's not necessarily when you look *back* and say, “Well that was a fantastic thing!” It's the day-to-day things that happen when you are really *doing* the experiments and you see the results and you say, “Wow, that's a great result!”

So from that way of looking at things, it's probably the earliest stuff that gave me the biggest kicks. When you are younger, you are probably more pleased by small results than when you get older. Those are really hard to recall, exactly, because they happen all the time! There are things on a weekly or monthly basis that give you a big kick.

**Gitschier:** You are lucky if that's true, actually. A lot of people have lots of failures weekly and monthly!

**Southern:** Well, I may be easy to please.

In my earlier career, I was a radiation chemist, and I had some very nice experiments in looking at the effects of radiation on proteins, actually polyaminoacids, which I used as a model for proteins. In that period, people wanted to find peaceful uses of atomic energy, and sterilization of food was one. Some of the discoveries I made then were, I think, really quite original. But it's a field that never went anywhere.

I was then very lucky to go into molecular biology in Edinburgh when I joined Peter Walker's group in 1967. It was quite late in my career; I was 29 at that time.

**Gitschier:** That doesn't sound too late to me, and I'm sure it doesn't sound too late to you anymore either!

**Southern:** No, you are right. The place where I was working made a decision to move out of Cambridge and to divide the lab into two. One group would go to Bristol and become a meat research institute, which had no appeal to me whatsoever.

**Gitschier:** Which, Bristol or the meat?

**Southern:** Either of those, actually. And the other would go to Norwich, which is better, and would become a food research institute.

But as it happened, a friend of mine was going to Edinburgh and he met Peter Walker, who was looking for somebody to join his group. So I had a chat with Peter and that was *it*, really. We hit it off.

It was an MRC group. Peter was a university professor, but he had his research group in the Department of Zoology. Pretty unusual for zoology in those days to be doing molecular work, but he had that sort of vision.

He was interested in what was called “satellite DNA”, and he gave me the job of sequencing mouse satellite DNA. One problem with that was that there weren't any sequencing methods at the time! I was very lucky that Ken Murray had just arrived in Edinburgh from Fred Sanger's lab, and he was working on the development of DNA sequencing methods. He basically held my hand and led me into DNA sequencing as he developed it.

It was guinea pig that gave me the first results, and speaking of which, going back to your question, seeing those fingerprints of the satellite DNA was such a moment, because you could see just instantaneously that it was a very simple sequence, much simpler than was thought by reassociation kinetics. That was a big moment for me.

At that time, the Murrays—Ken and Noreen—were working on restriction endonucleases and they introduced me to those fantastic enzymes. What a real tool those proved to be! We heard about Ham Smith's work on the type II restriction endonucleases. Ken had set up this club, and the rules were if you made a restriction enzyme, you could then have access to all the restriction enzymes that had joined the club, as it were. Edinburgh was a very lively place in those days, so there were plenty of people in the club.

It turned out that EcoRII gave this beautiful pattern with mouse satellite DNA—sort of a ladder pattern, which gave a huge amount of information about the structure of mouse satellite DNA at a glance. You could tell the repeat length, you could tell there was divergence of the sequence, you could measure the divergence of the ladder components, you could tell there were internal repeats within the major repeat, and so on. Just at a glance!

**Gitschier:** Just by ethidium bromide staining.

**Southern:** Yes! It was very simple. Just digest the DNA, run it on the gel, take a photograph, and that's it. And it was my colleague Gerard Roizes, who brought it out of the darkroom and said, “You have a ladder!”

**Gitschier:** Actually, I'm working on a book project about the '70s, and I'm provisionally calling it “The Thrill in a Dark Room.”

**Southern:** Well, there you go. You know, that was a really interesting period. There were so few labs working on genomic problems. There was Roy Britten at the Carnegie Institute of Washington, Brian McCarthy, an Englishman that worked in San Francisco, Don Brown at Carnegie. And then in Edinburgh there were Max Birnstiel and John Bishop, and there was also a famous epigenetics group that was set up by Conrad Waddington in the genetics department. He was a remarkable man, a real visionary.

**Gitschier:** I'm thinking even the word “epigenetics” seems ahead of its time. [According to Wikipedia, “epigenetics” was coined by Waddington in 1942 to describe the transition from embryonic state to differentiated state as a way of thinking about how the environment might influence phenotype during development.]

**Southern:** And in Edinburgh there was also Eric Reeves working on plasmids and Bill Hayes in the Department of Molecular Biology. We didn't appreciate it at the time, but looking back, it was a stunning collection of people. Some of the first recombinant DNA stuff was done there. Edinburgh was just an amazing place and still is.

So, where were we?

**Gitschier:** We're talking about the highlights along the way.

**Southern:** Yeah, the satellite DNA. The guinea pig sequence work established this idea that there is a *lot* of sequence in higher eukaryotes which couldn't possibly have a function that depended on its sequence; it couldn't be coding because it didn't make any sense.

I think that triggered in some people's minds that there was probably a lot of junk DNA. Francis Crick, Susumu Ohno, Peter Walker, all had that idea. And there is still a debate about that, of course, and I think the debate now is how much of the genome is junk, not the question of is there junk or not.

This business recently published, this ENCODE [Encyclopedia of DNA Elements] project, I don't know if you read any of it?

**Gitschier:** Well, I printed a few of them out.

**Southern:** Well done! Well, I was slightly depressed by the reports about it, because they spent *so* much on that. There are 400 or so authors on the papers—thirty-some papers. And the main conclusion seems to be, according to the newspapers, that there's not quite as much junk DNA as we thought there was! That's an awful lot of money to spend on that conclusion. No doubt, it will come.

So this notion of junk DNA came out of that work, largely from the guinea pig satellite DNA. But there is a nice irony here because a few years later, Bob Moyzis published the sequence of the human telomere, which turned out to have the same sequence as the guinea pig alpha-satellite. So, although I said firmly that there is no way this could be a functional sequence, actually there was a bit of mud on my boots there.

**Gitschier:** OK, I have a feeling we're now coming to blotting.

**Southern:** Right. That came out of a collaboration with Peter Ford, who was in the Department of Molecular Biology in Edinburgh at the time. Peter had studied the 5S RNA from frog oocytes. When the embryo starts its development, it synthesizes a huge amount of ribosomal RNA, but the 5S RNA has already been synthesized and stored. He had shown that the sequence of the 5S RNA in the oocytes is different from that in the somatic cells. That shows that there are two kinds of 5S genes, differentially regulated in frogs, with two different promoters. That was what everybody was interested in then: what are the eukaryotic promoters?

We thought it would be interesting to isolate the DNA from both kinds of genes for sequencing, and my thinking was we could use restriction enzymes to enrich for 5S genes. It was known that the genes were repeated, it was quite likely they would be tandemly repeated, like satellite DNA. The idea was that we would start with high molecular weight DNA and look for restriction enzymes that didn't cut the 5S genes, so we'd end up with a block of repeats that would stay close to the origin when you ran the stuff through a gel. You'd greatly enrich for the 5S genes, and once you'd done that, you could cut with enzymes that *did* cut the 5S, and you'd get a nice band of 5S genes.

But the problem that we were faced with is how did you know where the 5S genes are? We were cutting the gel up, eluting the DNA, and hybridizing with the 5S labeled RNA on [nitrocellulose] filters—the Gillespie and Spiegelman technique—that's how you measured genes in those days. And of course it was terribly noisy, and it's just a mess. So that was when I thought, “Well, if I could just hybridize the gel, then we might see a band.”

We tried drying the gels down and hybridizing to the gels, and that was very messy. Nothing came of it.

Then I thought about transferring it out of the gel onto cellulose nitrate paper, because Charles Thomas had developed this way of dissolving agarose gels in a high concentration of sodium perchlorate, a little known fact. My idea was to float the membrane on this 6M sodium perchlorate and then put the gel on top of it, and the sodium perchlorate would diffuse through the membrane, dissolve the gel, and the contents would sink and adsorb to the paper.

**Gitschier:** Was that part of that publication?

**Southern:** No. Because what happened was that as I was watching the thing sit and float in a boat, I saw a drop of liquid come through the gel on the top, through osmosis really, and that told me the gel was permeable. So if I just turned it upside down and put salt underneath the gel and put the membrane on top, it would soak the stuff through, which is what I did.

And that worked the first time, it was just so easy. That was another of those moments, a thrill in the dark room, as it were. This was quite early on, in 1973, actually.

**Gitschier:** But you didn't publish till '75.

**Southern:** I'm pretty slow to publish at the best of times, but I was rejected the first time I submitted it! I wanted it to go somewhere where it would get noticed. Methods papers tend to get buried, so I sent it to the *Journal of Molecular Biology*. Sydney Brenner was the editor and he rejected it because they didn't accept methods papers. But he said if you get some biologically relevant result, then we can publish it.

That delayed the publication, but in the mean time, we had a visitor in Edinburgh, Mike Mathews from Cold Spring Harbor. Mike saw all the things we were doing and asked me if he could use the method in his work, and I said “sure” and sketched out how to do it. He went back to Cold Spring Harbor, and they got it to work straight away and then showed it to visitors, and they all said, “Well, that's great, can *we* do it?”

I said it's fine to tell people as long as you tell them where it came from. So they were very honorable and did that. They acknowledged it as “Southern's technique”. So that is how my name got hung onto it.

**Gitschier:** Ah! I see. Now, at some point you start to think about using restriction enzymes to make a physical map of DNA.

**Southern:** I had the idea that we would establish a group that would have the remit of mapping the human genome. That idea really came from—this is going back to 1979—when I went to Woods Hole for the summer.

I wound up collecting all these bizarre creatures you can get in Woods Hole and extracting DNA from them, but the unfortunate thing was that when I came to look at the DNA, it was all degraded! Because I hadn't realized that the purified water was just filtered *seawater*! So all that experimental work was wasted, but I had a wonderful time.

While I was there, I had a visit from Dave Botstein, and I vividly remember this. He perched himself on the stool in the lab and told me that he wanted to use blotting to map the human genome. He had this idea of using restriction enzymes and these restriction enzyme polymorphisms, and so on. I didn't understand what he was talking about. It made no sense. But he was *really* enthusiastic about it, so I thought, “Well there must be something in this!”

So, when I went back, I thought well, the obvious thing to do, alongside the genetic map, is to make a physical map. And I had ideas about how to do that: cosmid libraries, fingerprinting, that sort of thing.

And then there was a lot of talk about sequencing the genome. Sydney Brenner got an interest in the whole area. Sydney and I were pushing the idea of just sequencing the coding regions first of all.

But actually it was Fred Sanger who should get the credit for this genomic sequencing idea, because Fred not only developed a sequencing method, he also developed this idea that you just start with a genome and you sequence it completely. He started with phiX174 genome and just sequenced the whole of that. Out of that came things like alternative codons and so on. Then, mitochondrial DNA, he sequenced that. And then, he went on to phage lambda and sequenced that. He just went to more and more complex genomes and without any original hypothesis. He said, “Well, let's just sequence the whole thing and see what comes out of it.”

John Sulston, of course, was a colleague of Fred's, and he went on to sequence the nematode genome. It was an extraordinarily courageous thing to do, I think. But he was determined and pushed it through. He was then pushing for sequencing the whole human genome. That became the UK effort.

There was a lot of talk in the mid-'80s that Sanger's method was too expensive. I went to a meeting in Japan, organized by Akiyoshi Wada specifically to answer the question of whether there is a quicker, cheaper way than Sanger sequencing.

Wada himself gave a paper. A colleague of his had shown that if you put an oligonucleotide on a column and pass a tRNA down it, then you can fractionate, essentially, a nucleic acid that differs by a single mismatch. I'm not sure that he said this, but basically, you are reading a bit of sequence by doing that. If a nucleic acid sticks to the column under the conditions they were using, then you'd essentially read that bit of sequence of the stuff that sticks to the column. So it occurred to me that if you had a complete set of all oligonucleotides—every sequence of a given length, each one in a column—then you could sequence. I can remember talking to George Church about it while hanging onto a strap on a bus. That was one of those moments, having the first idea that if you had a lot of oligonucleotides, this was essentially a sequence reading tool. That was very pleasing.

I sat down and thought about that and realized that that would be an awful lot of columns! Because you know, if you chose octanucleotides, then there are 65,536 different octanucleotides.

**Gitschier:** That's a lot of columns!

**Southern:** But then I thought, if you had these on a spot on a membrane, you could do it that way. You'd label up your genomic DNA and you'd hybridize it to this piece of paper or whatever kind of matrix you had.

So I started looking at the chemistry for making oligonucleotides. Beautiful chemistry: the reactions are quick, they go to 99%, and it's done on glass! This wonderful material called controlled pore glass, a mixture of ordinary glass and borosilicate glass, in which all the silicate glass is etched away, leaving this mesh of borosilicate glass with a massive surface area.

Finding out about the chemistry was a real thrill, as well, and then realizing we could very simply adapt the chemistry for making oligonucleotides to print them on glass. With a German PhD student, Uwe Maskos, we developed a way to leave them attached to the glass. And that worked beautifully. It showed that you could synthesize oligonucleotides on the glass at high yield, that they would hybridize, and off we went. We worked on different ways of making arrays of different sequences, and then moved from there into applications. We thought that sequencing would be an application, but we didn't have the means to make the size of arrays that you would need for sequencing.

In 1987 I wrote a grant application to the MRC to develop this methodology. And that was successful, but at the same time the university was pressing us to patent any new ideas. I was on the committee, actually, that set up this group to exploit intellectual property. So they came to me and said, “Do you have any ideas about a patent?” And the grant application became a patent application.

And that's been a long story.

**Gitschier:** In that it's been so successful?

**Southern:** Yes. But we had a few battles along the way.

**Gitschier:** Battles in terms of patent infringements.

**Southern:** Yeah. This was the first patent that the university's tech transfer company had wanted to take through exploitation. And they went into a license agreement with Beckman, but really, it wasn't part of Beckman's mainstream interests. So eventually we brought the licensing back to the university.

And then the university weren't being very energetic about licensing it, and there were people out there who were infringing it, I thought. If you have a patent and somebody's infringing it, the only recourse you have is to sue! And suing is an extremely expensive business. It's not what a university should be spending their time and energies on. I had to make a decision. Do I want to just let this thing go? Or carry on with it?

I decided to carry on with it. I negotiated with the university to take it and set up my own company and to assign a license to my own company, Oxford Gene Technology. And then we got into this whole business of licensing and litigation and so on. We sublicensed to a lot of people; Agilent and Affymetrix have a lifetime license.

That licensing gave me quite a substantial income, and that gave me opportunities, then, to develop other things. It helped set up OGT in a more substantial way, and I set up two trusts, the Kirkhouse Trust and the Edina Trust.

When I first set up Kirkhouse Trust, my idea was to go into medical areas that I was familiar with. But Paul Nurse, who is now President of the Royal Society, was a trustee and he said, “That is such a crowded area, why don't you do something different?”

And so I said, “Well, what have you got in mind?”

And he said, “Well, what about crops?”

And I said, “Well, I don't know anything about crops!”

And he said, “Good reason to get going!”

**Gitschier:** Well, did *he* know anything about crops?

**Southern:** Only in a general way. I mean, his first degree is in botany.

What we [Kirkhouse Trust] do is decide on a crop species, and we gather together a group of breeders across a region. We're in West Africa for a crop called cowpea, which you would call a black-eyed pea, and then in East Africa for common bean, which is kidney bean or navy bean. We go to those regions with somebody knowledgeable in the area, from the States usually. We have Paul Gepts from UC [University of California] Davis and Mike Timko from UVA [University of Virginia]. They give the leadership to the whole thing because they are molecular geneticists, as well as plant breeders.

We find the crop breeders who are working on those crops in the region and bring them together into a consortium, and we say, “We want to help you establish molecular techniques in your breeding programs.” So we set up basic labs in Africa. We give them equipment for doing DNA extraction, PCRs, running gels, taking gel photographs, interpreting the data, and we train the people in those methods. The effect of that is remarkable; all kinds of things fall out. You can look at the diversity of the crops, which is an important first step in any breeding program, but you can also track the gene through the generations of the breeding program in a very robust way.

We're interested in resistance to pests and diseases. And remarkably, there are a *lot* of these genes already present in the species. Take the bean, for example, we're working on five different “constraints” [pests or diseases], as they are called. And there are resistances known to all of those, mainly coming from South America, where beans originated. And with the molecular techniques you can combine resistances. That is really difficult unless you use the molecular methods, tracking the genes. It speeds things up enormously and it's working!

We already have cowpeas that we developed in West Africa that are out in the farmers' fields! There, the main pest is a parasitic weed called Striga, or witchweed. This is a parasite that latches itself onto the outside of the roots of the cowpeas and sucks out all of the goodness, and the cowpea dies. And there is very little you can do about it once it's established. On average, it destroys about 30% of the crop.

Two hundred million people in West Africa are dependent on this crop for their source of high-quality protein. In some years, 100% of the crop is destroyed by this weed. Very quickly, within 3 years, we were able to establish resistant varieties. There are seven different races of Striga, and each one has a different resistance gene associated with it, apart from one.

**Gitschier:** I think one of things that is so interesting about life is that you can't always anticipate where it is going to lead you. This is clearly an example for you.

**Southern:** You couldn't say that I had a planned career. It's just evolved. And I've followed things as they have arisen, and I think that is the best way to go. I've certainly ended up in places where I've never imagined.

**Gitschier:** I'm just wondering. If you could just start over today, do you have any idea what you might want to do?

**Southern:** Yeah. I'd be an engineer. Definitely!

**Gitschier:** What kind of engineer?

**Southern:** Well, I think a lot of the problems that we are faced with have engineering solutions, for example, in new energy systems, there are tremendously interesting engineering problems. That's what I would want to do.

I like DIY [do it yourself], building things in the lab, that sort of thing. Engineering brings a lot of things together. It's mathematical skills and imagination. That's what it needs and it must be tremendous fun, I think, to see your ideas made into something that works.

